# Plasma Purification Treatment Relieves the Damage of Hyperlipidemia to PBMCs

**DOI:** 10.3389/fcvm.2021.691336

**Published:** 2021-07-07

**Authors:** Xiao Meng Zhang, Yan Hong Gu, Hao Deng, Zheng Quan Xu, Ze Yuan Zhong, Xia Jie Lyu, Hui Min Jin, Xiu Hong Yang

**Affiliations:** ^1^Division of Nephrology, Pudong Medical Center, Shanghai Pudong Hospital, Fudan University, Shanghai, China; ^2^The First Affiliated Hospital of Medical School of Zhejiang University, Zhejiang University, Hangzhou, China; ^3^Division of Orthopedic, Pudong Medical Center, Shanghai Pudong Hospital, Fudan University, Shanghai, China; ^4^School of Basic Medicine, Weifang Medical University, Weifang, China

**Keywords:** plasma purification, PBMC, lipid metabolism, endoplasmic reticulum stress, ROS

## Abstract

**Background:** Hyperlipidemia {hypercholesterolemia [cholesterol >5.18 mmol/L) or hypertriglyceridemia [triglycerides >2.3 mmol/L], mixed hyperlipidemia [cholesterol >5.18 mmol/L and triglycerides >2.3 mmol/L], and high low-density lipoproteinemia [low-density lipoprotein (LDL) >3.4 mmol/L]} is a strong risk factor for arteriosclerosis and cardiovascular disease (CVD). Therapy with lipid-lowering drugs often results in many side effects. Our study aimed to investigate the potential effects of non-drug therapy with double-filtration plasmapheresis (DFPP) on lipid metabolism-, endoplasmic reticulum (ER) stress-, and apoptosis-related proteins in peripheral blood mononuclear cells (PBMCs) before and after lipid clearance in patients with hyperlipidemia.

**Methods:** Thirty-five hyperlipidemia patients were selected. Proteins related to lipid metabolism [CD36, proprotein convertase subtilisin/kexin type 9 (PCSK9), and LDL receptor], ER stress [glucose-regulated protein 78 (Grp78), C/EBP homologous protein (CHOP), activating transcription factor 4 (ATF4), and eukaryotic initiation factor 2α (EIF2α)], and apoptosis [B-cell lymphoma-2 (Bcl-2), Bcl-2-associated X protein (BAX), and cysteinyl aspartate specific proteinase-3 (Caspase-3)] were assayed by Western blot, reactive oxygen species (ROS) were measured by flow cytometry (FCM), and ELISA detected serum inflammatory [interleukin (IL)-1β, IL-6, and tumor necrosis factor α (TNF-α)] factors.

**Results:** Compared with their pre-DFPP values, the values of most lipid metabolic parameters, such as cholesterol, triglycerides, LDL, lipoprotein a [Lp(a)], and small dense LDL (sdLDL) cholesterol, were reduced after DFPP. DFPP was associated with the downregulation of proteins related to lipid metabolism, ER stress, and apoptosis, resulting in decreased ROS and serum inflammatory factor release.

**Conclusion:** DFPP has lipid-lowering activity and can also regulate lipid metabolism-, ER stress-, and apoptosis-related proteins in PBMCs and reduce the levels of inflammatory factors in patients with hyperlipidemia (ClinicalTrials.gov number: NCT03491956).

## Introduction

Hyperlipidemia is one of the most important factors associated with cardiovascular disease (CVD) and often results in fatty liver, cerebral thrombosis and/or infarction, and severe pancreatitis (1–4). Lipid disorders are present in 40.4% of the adult population in China, with hypercholesterolemia present in 4.9% of the population, hypertriglyceridemia present in 13.1% of the population, and high low-density lipoproteinemia [low-density lipoprotein (LDL)] present in 33.9% of the population (5). Therefore, there is an urgent need for prevention and treatment of increased serum lipid concentrations.

A critical marker of hyperlipidemia is an abnormal expression of lipid-related metabolic proteins. CD36 is a lipid transporter and multifunctional scavenger receptor (6). It plays roles in the modification of LDL and uptake of modified lipoproteins, which causes macrophage lipid accumulation, leading to the formation of foam cells (7). In hyperlipidemia patients, CD36 expression was abnormal, and increased CD36 expression could result in endoplasmic reticulum (ER) stress, macrophage apoptosis, insulin resistance, and CVD (8). Another important lipid metabolic protein, proprotein convertase subtilisin/kexin type 9 (PCSK9), is mainly expressed in the liver, intestinal tract, and kidney (9). In the context of decreased PCSK9 activity, LDL receptor (LDLR) is upregulated, resulting in a decrease in LDL cholesterol levels, potentially reducing the risk of CVD (10). A previous study has indicated that PCSK9 levels were associated with metabolism-related indices, such as fasting blood glucose, insulin, triglycerides, and liver triglyceride content (11). Alirocumab, a PCSK9 inhibitor, has been approved by the US Food and Drug Administration (FDA) in the treatment of patients with hypercholesterolemia. In another study, Alirocumab was found to reduce or improve cardiovascular outcomes after acute coronary syndrome (12). A previous study has indicated that PCSK9 and CD36 are associated with lipid metabolism and CVD outcomes. New pharmacological agents are under development to lower the risk of CVD by inhibiting PCSK9 (13). However, it is unclear whether lipid plasmapheresis could affect CD36, PCSK9, and LDLR.

Statins are the most frequently used drug for the treatment of hypercholesterolemia. However, adverse reactions, such as rhabdomyolysis, abnormal liver enzymes, and gastrointestinal symptoms, frequently prevent the long-term use of this medication. Moreover, a meta-analysis indicated that statins were associated with an increased risk of insulin resistance and new-onset diabetes (14). Some patients are reluctant to take lipid-lowering drugs considering the adverse effects of statins, and some are worried about the control of blood glucose, especially among patients with diabetes.

Non-drug therapy to reduce lipid levels has been used for many years. Heparin-induced extracorporeal LDL precipitation (HELP) was proven to be capable of reducing lipid levels and the risk of CVD in patients with familial hypercholesterolemia in a previous study; however, the high costs of consumption and treatment prevented its further large-scale clinical usage. Instead, double-filtration plasmapheresis (DFPP) has recently replaced HELP in treating familial hypercholesterolemia, hyperlipidemia-induced severe acute pancreatitis, and coronary artery disease after coronary artery stenosis (15–18). DFPP has the advantages of easy use for operators, safety for patients, a low price (7,000 RMB, equal to US$1,000/session, can be covered by Medicare), a short treatment time (3–4 h), and no plasma or albumin replacement. Combined with clinical observations, we recommend that patients with hyperlipidemia undergo regular rechecks, routinely receive DFPP treatment every 6 months, and pay attention to diet and healthy routines after treatment. We have observed that in addition to the decline in lipid profiles after treatment, patients have many interesting effects, such as alleviating cervical discomfort, “tired eyes,” “blurry eyes,” “stiff shoulders,” and “feeling of ill-health.”

DFPP technology has been used to treat hyperlipidemia patients at Pudong Medical Center since 2017. The study aimed to investigate the lipid removal effect of DFPP and the association of DFPP with changes in lipid metabolism- and ER stress-related proteins in peripheral blood mononuclear cells (PBMCs) before and after treatment in patients with hyperlipidemia.

## Materials and Methods

### Study Participants

Patients who had hyperlipidemia [diagnosed with hypercholesterolemia (cholesterol >5.18 mmol/L) or hypertriglyceridemia (triglycerides >2.3 mmol/L), mixed hyperlipidemia (cholesterol >5.18 mmol/L and triglycerides >2.3 mmol/L), and high low-density lipoproteinemia (LDL >3.4 mmol/L) according to the 2019 guidelines of the European Society of Cardiology] with no other diseases were selected for this study. No statins or other lipid-lowering drugs were prescribed for the patients. All patients provided and signed informed consent. The Ethical Committee approved the study of Shanghai Pudong Hospital (Fudan University, Pudong Medical Center, Shanghai, China), and experiments were performed in accordance with the Ethical Committee's guidelines and regulations. The ClinicalTrials.gov number was NCT 03491956.

#### Exclusion Criteria

Patients were excluded if (1) they had coexisting diabetes or hypertension; (2) they had tumors, liver and kidney dysfunction, severe edema, cardiac insufficiency, respiratory insufficiency caused by severe lung disease, or pregnancy; and (3) they were over 80 years old.

### Treatment Procedure

Plasmapheresis using a Plasauto iQ automatic blood purification system, model KM-9000 (Kawasumi, Tokyo, Japan), was performed in this study, with a PE-08 primary membrane plasma separator and an EC-4A20 secondary membrane plasma component separator. The cubital elbow median veins on both arms were selected for artery and vein access. The blood flow was 60–100 ml/min, the plasma separation rate was 30% of the blood flow, and the plasma rejection rate was 15% of the plasma separation rate. Standard heparin was used as an anticoagulant, with an initial dose of 3,000 U, followed by an additional maintenance dose of 20–40 U/(kg·h). The therapeutic target was calculated as weight (kg) × 40 ml (1–1.4 times blood volume). Each session lasted for 3–4 h. Lipid profile is tested by the Department of Laboratory Medicine, Pudong Hospital.

### Laboratory Examination

#### Isolation of PBMCs

Blood samples were taken from patients with hyperlipidemia, and acidic citrate dextrose (Walvax Biotechnology Co., Yunnan, China) was added as an anticoagulant. To isolate PBMCs, the blood samples were diluted with phosphate-buffered saline (PBS), and PBMCs separation medium was added (Hao Yang Biological Manufacture Co., Tianjin, China). The samples were centrifuged at 1,000 rpm for 40 min, the middle cell layer was collected, and PBMCs were obtained after washing three times with PBS.

#### Detection of Lipid Metabolism-, ER Stress-, and Apoptosis-Related Proteins

PBMCs protein samples are extracted from patients with high cholesterol and/or high LDL cholesterol (LDL-C). To ensure the experimental results' accuracy, all protein samples will be tested immediately after the patient's treatment is completed. The proteins subjected to Western blotting were extracted using lysis buffer (Invent, Beijing, China), separated by sodium dodecyl sulfate-polyacrylamide gel electrophoresis (SDS-PAGE), and transferred onto polyvinylidene difluoride (PVDF) membranes (Millipore, MA, USA). The membrane carrying the protein bands was blocked in Tris-buffered saline with Tween (TBST) containing 5% skimmed milk for 1 h and incubated with primary antibodies at 4°C overnight. After washing, the membrane was incubated with secondary antibodies conjugated to horseradish peroxidase for 1 h at room temperature. Secondary antibodies included goat anti-rabbit IgG and goat anti-mouse IgG. After washing, the membranes were incubated with Immobilon Western Chemiluminescent HRP Substrate (Millipore, MA, USA). Protein signals were captured using a Bio-Rad ChemiDoc™ XRS system (Bio-Rad, CA, USA). Data were quantified by Quantity One software (Bio-Rad, CA, USA) (19). Primary antibodies against LDLR (Cat No. 10785-1-AP), CD36 (Cat No. 18836-1-AP), PCSK9 (Cat No. 55206-1-AP), glucose-regulated protein 78 (GRP78; Cat No. 11587-1-AP), C/EBP homologous protein (CHOP; Cat No. 15204-1-AP), activating transcription factor 4 (ATF4; Cat No. 10835-1-AP), p-EIF2α (Cat No. 28740-1-AP), B-cell lymphoma-2 (Bcl-2; Cat No. 12789-1-AP), Bcl-2-associated X protein (BAX; Cat No. 50599-2-Ig), and cysteinyl aspartate specific proteinase-3 (Caspase-3; Cat No. 19677-1-AP) and secondary antibody goat anti-rabbit IgG (1:2,000) were obtained from Proteintech (Proteintech Group, Inc., IL, USA), and an antibody against eukaryotic initiation factor 2α (EIF2α; Cat No. 9722S) was purchased from CST (Cell Signaling Technology, MA, USA). The antibodies are diluted according to the instructions, LDLR (1:2,000), CD36 (1:1,500), PCSK9 (1:2,000), GRP78 (1:2,000), CHOP (1:2,500), ATF4 (1:2,000), p-EIF2α (1:2,000), Bcl-2 (1:5,000), BAX (1:2,000), and Caspase-3 (1:5,000). Data were quantified by the Quantity One software (Bio-Rad).

#### Detection of ROS and Serum Inflammatory Factors

Non-stimulated PBMCs were detected with a ROS assay kit (Beyotime, Shanghai, China) after isolation. The level of reactive oxygen species (ROS) was determined based on the oxidative conversion of cell permeable 2′,7′-dichlorofluorescein diacetate (DCFHDA) to fluorescent dichlorofluorescein (DCF) upon reaction with hydroxy radical, hydrogen peroxide, or peroxynitrite. PBMCs in each sample were washed twice with PBS. The fluorescent probe used to determine ROS's level in PBMCs was diluted to a concentration of 10 μmol/L. PBMCs were suspended in the diluted fluorescent probes and incubated in a plate at 37°C for 20 min. The PBMCs were washed three times with cell culture medium to remove probe that did not enter the cells. Finally, the ROS level was detected by flow cytometry (FCM; BD, NH, USA) with excitation and emission settings at 488 and 525 nm, respectively, and expressed as a percentage of pre-DFPP. Data were analyzed by the FlowJo software (BD, NH, USA). Serum inflammatory factors [interleukin (IL)-1β, IL-6, tumor necrosis factor α (TNF-α)] were detected by ELISA using commercial kits (Neobioscience, Shanghai, China).

### Statistical Analysis

Data are expressed as mean ± SD, except skewed data that are expressed as median (range). Paired *t*-test and Wilcoxon rank-sum tests are used to analyze the data, and a *P* < 0.05 was considered statistically significant. Statistical analyses were performed using SPSS 22.0 statistical software (IBM, IL, USA).

## Results

### Clinical Characteristics of the Patients

The age, sex, weight, and baseline lipid profiles of the patients are shown in Table 1. The mean serum total cholesterol post-DFPP was 2.4 [1.8–3.1] mM, which represented a significant decrease compared with the pre-DFPP values. Serum LDL-C and triglycerides post-DFPP showed the same trend. Compared with the values measured before DFPP, Lp(a), and sdLDL values also showed an obvious decrease post-DFPP. These data indicated that DFPP could remove blood lipid molecules effectively in 3–4 h.

**Table 1:** The variations of lipids before and after DFPP treatment.

	**Pre-DFPP (*N* = 35)**	**Post-DFPP (*N* = 35)**
Age (years)	51 [44, 61]	–
Male	12	–
Weight (kg)	73.1 [68.4, 83.9]	–
Total cholesterol (mM)	8.2 [6.6, 10.0]	2.4 [1.8, 3.1]^**^
Triglyceride (mM)	8.9 [7.1, 10.2]	2.1 [1.5, 3.1]^**^
LDL-C (mM)	4.7 [3.9, 5.5]	1.4 [1.1, 1.8]^**^
Lp(a) (mg/L)	187.3 [137.9, 200.7]	78.3 [53.8, 104.8]^**^
sdLDL-C (mM)	2.1 [1.8, 2.7]	0.3 [0.2, 0.5]^**^
Patients with hypercholesterolemia	18	–
Patients with hypertriglyceridemia	10	–
Patients with mixed hyperlipidemia	3	–
Patients with high low-density lipoproteinemia	4	–

*Pre-DFPP, patients before DFPP treatment; Post-DFPP, patients after DFPP treatment; LDL-C, low density lipoprotein; Lp(a), lipoprotein(a); sdLDL, small dense low-density lipoprotein. Data were expressed as median (range)*.

** *P < 0.01 vs. pre-DFPP*.

### DFPP Is Associated With Changes in Lipid Metabolism-Related Proteins

**Figure 1:**
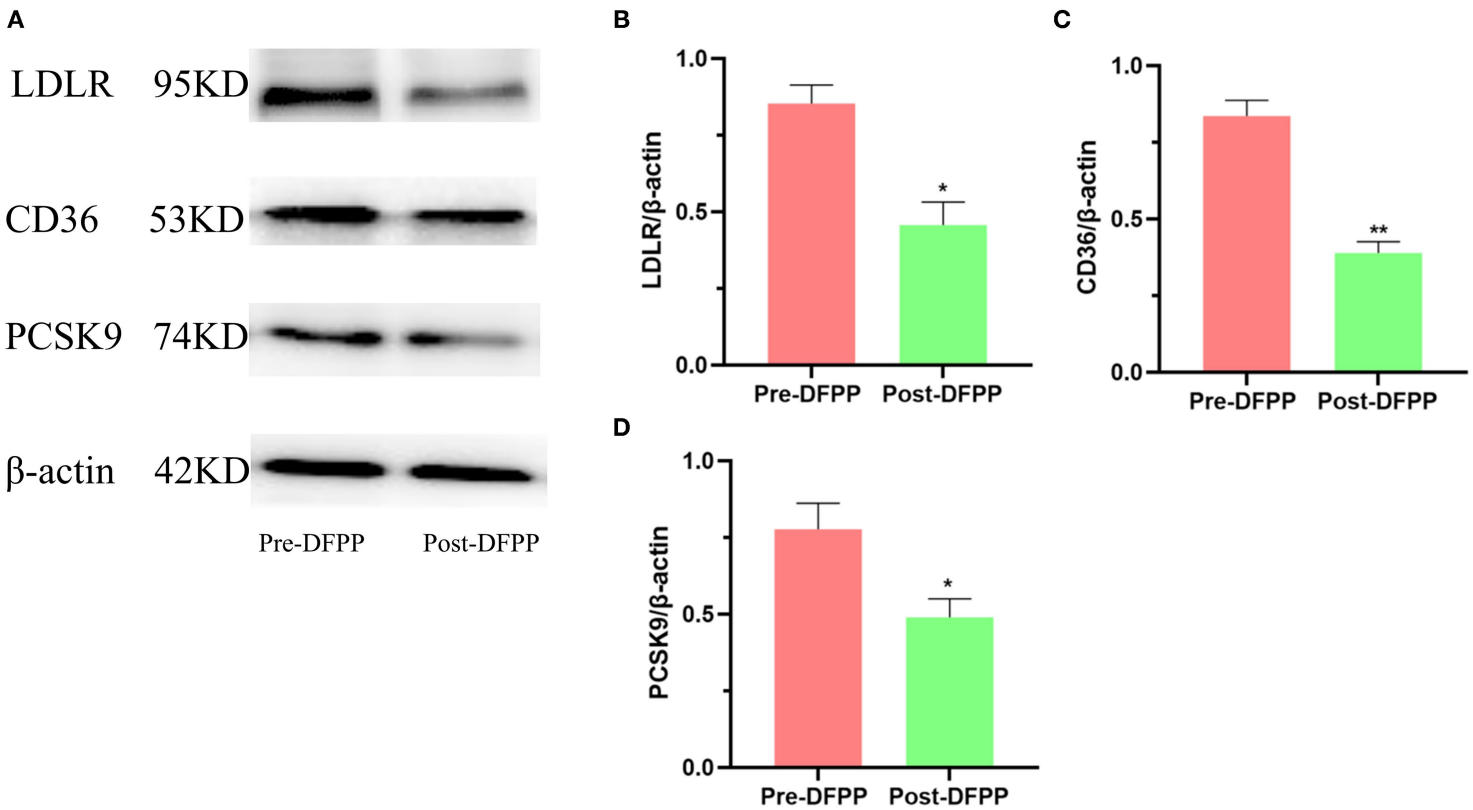
Effects of DFPP on expressions of lipid metabolic proteins. PBMCs protein samples are extracted from patients with high cholesterol and/or high LDL-C. **(A)** Westernblot result picture of lipid metabolic proteins. Western blot analyses detected the protein expression of LDLR **(B)**, CD36 **(C)**, and PCSK9 **(D)** in PBMCs. Pre-DFPP: patients before DFPP. Post-DFPP, patients after DFPP. Values were expressed as mean ± SD. *P < 0.05 and **P < 0.01 vs. pre-DFPP. *N* = 5.

PBMCs were separated from blood samples in time, and protein samples were also extracted from PBMCs in time. The protein expression levels of PBMCs lipid metabolism-related proteins assayed by Western blot are shown in Figure 1. LDLR, PCSK9, and CD36 were all downregulated post-DFPP. These indicators are closely related to CVD occurrence. Our results indicated that DFPP may influence key lipid metabolism-related transport proteins.

### DFPP Reduces the Expression of ER Stress-Related Proteins

**Figure 2:**
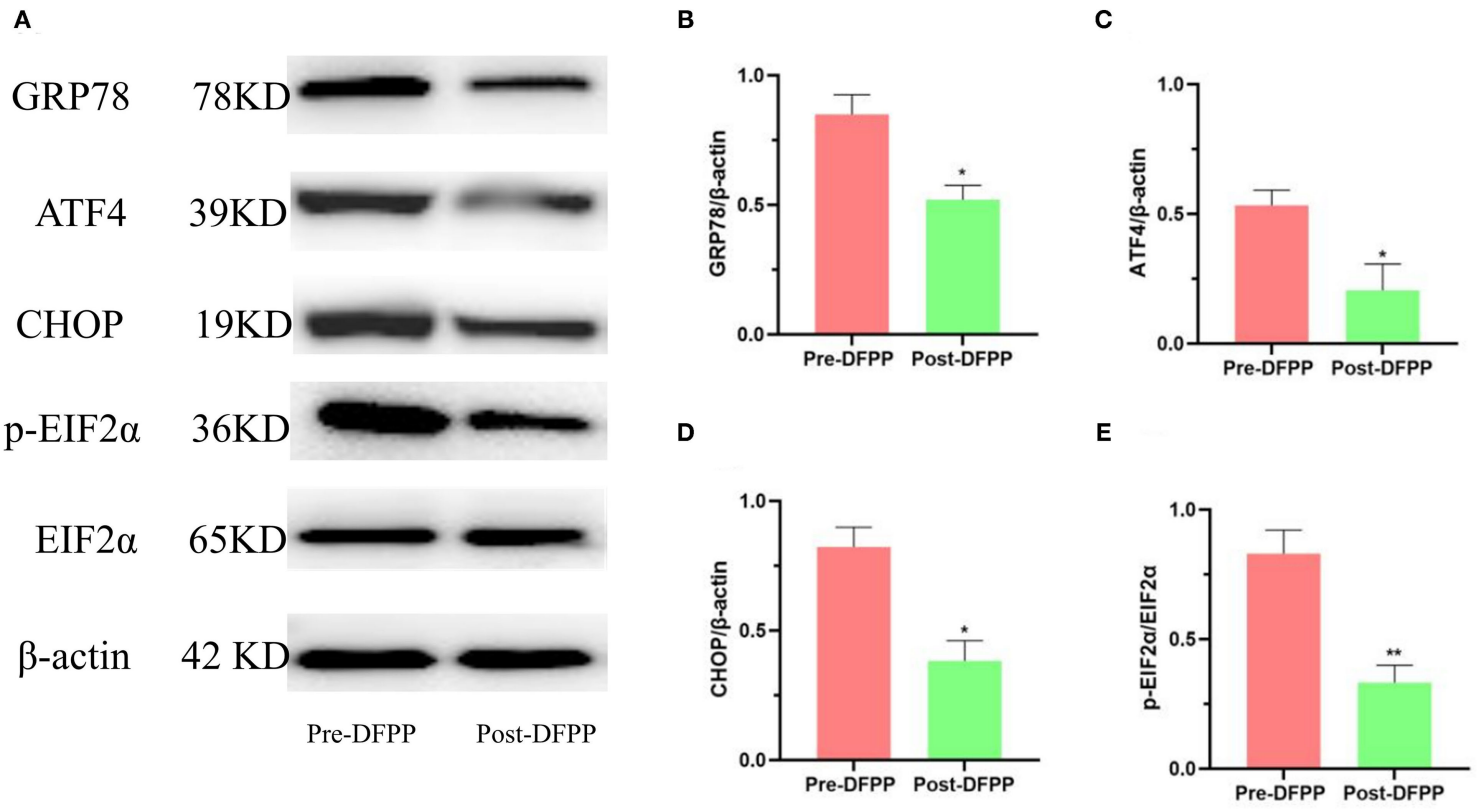
DFPP affects ER stress-related proteins. PBMCs protein samples are extracted from patients with high cholesterol and/or high LDL-C. **(A)** Westernblot result picture of ER stress-related proteins. ER stress-related proteins [GRP78 **(B)**, ATF4 **(C)**, CHOP **(D)**, p-EIF2α **(E)**] in PBMCs were detected by Western blot analyses. Values were expressed as mean ± SD. *P < 0.05 and **P < 0.01 vs. pre-DFPP. *N* = 5.

This part of the experiment also detected PBMCs protein samples. The expression of ER stress-related proteins (GRP78, CHOP, ATF4, and p-EIF2α) is shown in Figure 2. Compared with the expression of these proteins prior to DFPP, GRP78, CHOP, ATF4, and p-EIF2α showed significant decreases post-DFPP. These proteins are the marker proteins of ER stress. As the expression level of these proteins decreased, the ER stress decreased. These data demonstrated that DFPP was associated with reduced ER stress in hyperlipidemia patients.

### DFPP Inhibits Hyperlipidemia-Induced Cell Apoptosis-Related Protein Expression

**Figure 3:**
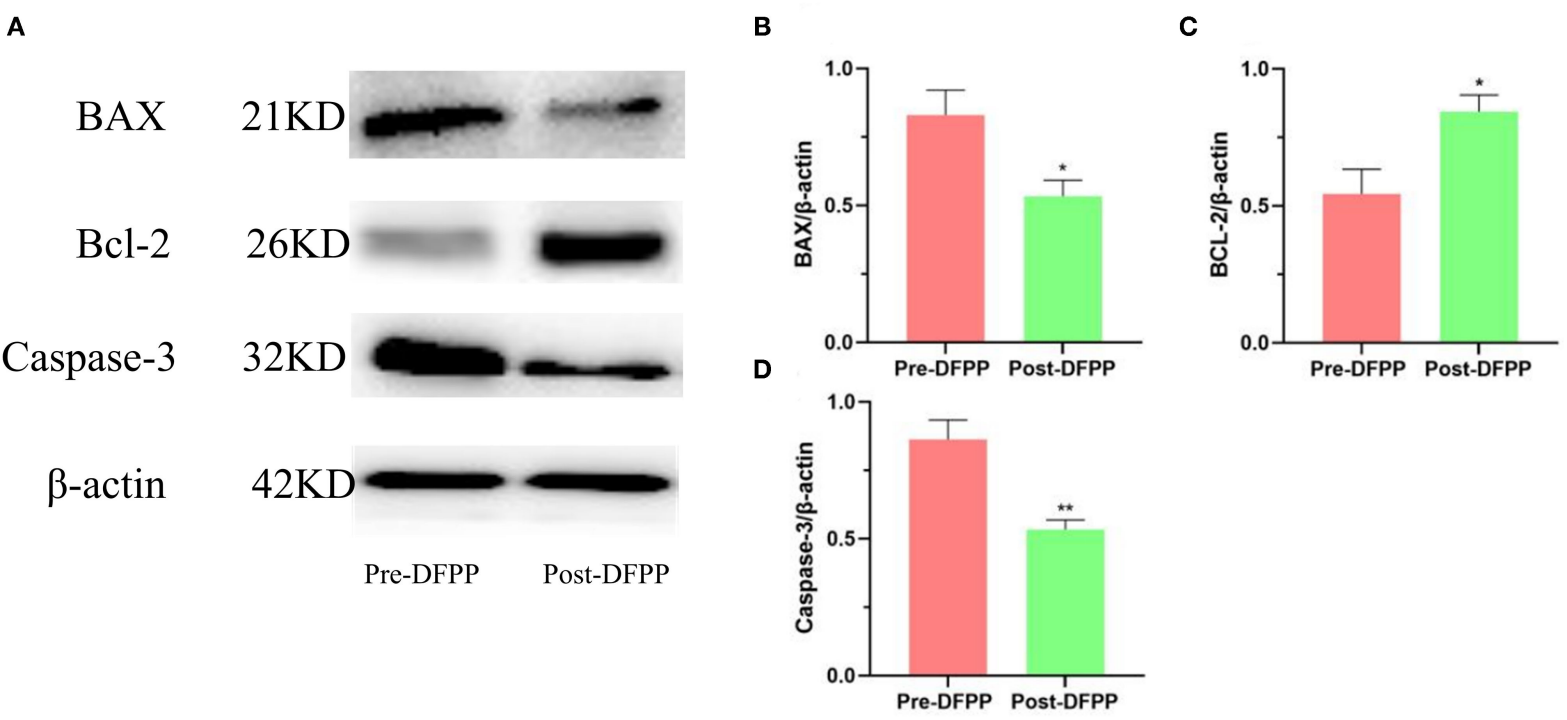
Effects of lipid plasmapheresis on expressions of apoptosis-related proteins. PBMCs protein samples are extracted from patients with high cholesterol and/or high LDL-C. **(A)** Westernblot result picture of apoptosis-related proteins. Apoptosis-related proteins [Bcl-2 **(B)**, BAX **(C)**, Caspase-3 **(D)**] in PBMCs were examined by Western blotting analyses. Values were expressed as mean ± SD. *P < 0.05 and **P < 0.01 vs. pre-DFPP. *N* = 5.

The protein samples of the patient's PBMCs before and after treatment were detected by Western blot. As Figure 3 shows, apoptosis-related proteins have changed significantly after DFPP treatment. The expression of BAX and Caspase-3 both decreased significantly, and Bcl-2 increased significantly. The above results showed that DFPP treatment changed the expression of the apoptotic protein. Compared with before treatment, PBMCs apoptosis after treatment is inhibited.

### DFPP Reduces ROS Levels in PBMCs and Alleviates Inflammatory Factors in Serum

**Figure 4:**
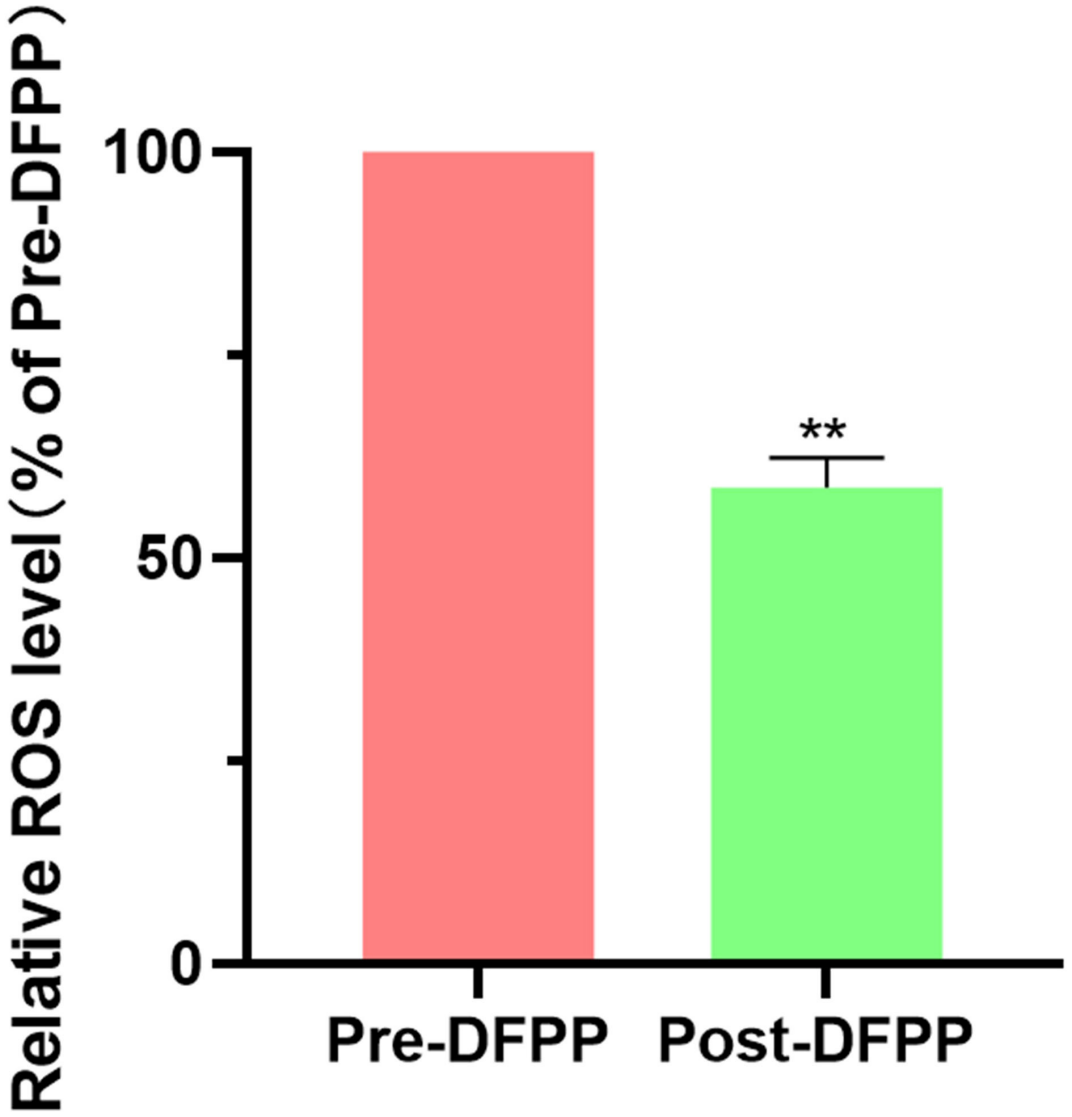
DFPP reduces ROS level. PBMCs samples are extracted from patients with high cholesterol and/or high LDL-C. PBMCs. ROS levels were measured by flow cytometry before and after DFPP treatment. PBMCs were suspended in the diluted fluorescent probes and incubated in a plate at 37°C for 20 min. The result was expressed as a percentage of pre-DFPP. Values were expressed as mean ± SD. **P < 0.01 vs. pre-DFPP. *N* = 5.

The ROS levels in PBMCs from hyperlipidemia patients were significantly decreased compared with those of patients before DFPP (Figure 4). In addition, in Table 2, markers of inflammation showed an obvious decline after DFPP. These results showed that DFPP could effectively decrease ROS levels (~43%) and reduce serum inflammatory factor release in hyperlipidemia patients.

**Table 2:** DFPP reduces inflammation parameters.

	**Pre-DFPP (*N* = 35)**	**Post-DFPP (*N* = 35)**
IL-1β (pg/mL)	17.9 [15.3, 20.1]	9.3 [8.1, 10.7]^*^
IL-6 (pg/mL)	16.0 [13.4, 20.8]	5.6 [4.1, 8.4]^**^
TNF-α (pg/mL)	21.7 [19.5, 23.5]	9.3 [8.5, 11.0]^**^

*Pre-DFPP, patients before DFPP treatment; Post-DFPP, patients after DFPP treatment; Serum IL-6, TNF-α, IL-1β in patients with hyperlipidemia were detected by ELISA kit. Data were expressed as median (range)*.

* *P < 0.05 and*

** *P < 0.01 vs. Pre-DFPP*.

### Adverse Events

One patient experienced a hypotensive episode during the procedure and quickly recovered after infusion of 0.9% normal saline. No other adverse events (blood coagulation, bleeding, or allergy) occurred during the treatment period.

## Discussion

Our study indicates that DFPP can effectively reduce the levels of lipids, such as LDL-C, cholesterol, triglycerides, Lp(a), and sdLDL cholesterol, a finding similar to that of a previous study (15). The safety, easy operation, and low cost of DFPP indicate that it is suitable for widespread clinical use to reduce hyperlipidemia markers.

Patients with hyperlipidemia often exhibit abnormal expression of lipid metabolic proteins. CD36 expression was increased in patients with hypercholesterolemia (20). A previous study showed that CD36 promoted free fatty acids' cellular uptake and regulated intestinal cholesterol synthesis (21). Additionally, oxidized low-density lipoprotein (oxLDL) induced platelet activation and ROS production, ultimately resulting in thrombosis via the CD36 and MAPK pathways (22–24). An *in vivo* study showed that insulin resistance and inflammatory factor release were reduced in CD36-knockout mice, indicating that CD36 plays a vital role in humans (25). CD36 is closely related to PCSK9. One of the PCSK9 targets for degradation is CD36. Furthermore, PCSK9 and CD36 play an important role in triglyceride metabolism (26). In CVDs, there is a piece of evidence that PCSK9 is related to myocardial infarction caused by platelet activation through CD36 while these factors are found in various organs and behave differently (27). When PCSK9 was inhibited, CD36 expression increased in the intestine but decreased in the liver and glomerular podocytes (26). PCSK9 itself is a member of the proprotein invertase family of proteins, which regulate various physiological and pathological processes, including lipid metabolism, inflammatory responses, glucose metabolism, apoptosis, and other processes (28). PCSK9 is an important player in cholesterol homeostasis. By binding to hepatic LDLR and promoting its degradation, PCSK9 reduces LDL-C uptake, leading to an increase in plasma LDL-C concentrations. In previous research, it was found that PCSK9 might impair the function of LDLR. Deleting the *LDLR* gene in familial hypercholesterolemia may be an important event in the disease's pathogenesis (29). A previous study showed that lipid plasmapheresis reduced plasma PCSK9 levels (30). Similarly, our study showed that lipid plasmapheresis induced the downregulation of the PCSK9 protein in isolated PBMCs. For the first time, the protein expression of CD36 and LDLR was shown to be obviously downregulated in PBMCs after lipid plasmapheresis. This result differs from the result obtained after statin therapy. In patients receiving statin therapy, PCSK9 and LDLR were upregulated, and CD36 was downregulated (31). Besides, PCSK9 is a secreted protein from the liver, kidneys, lungs, etc. In this experiment, other tissue samples could not be obtained for further research due to ethical reasons. There is no research on PCSK9 in PBMCs, and no direct evidence to prove the source of PCSK9 changes in DFPP treatment. A previous study has observed an increase in the serum concentration of PCSK9 in patients with acute coronary syndrome, a higher level of LDLR on the surface of monocytes, and an increase in LDLR mRNA transcription (32). Therefore, we speculate that DFPP reduces the PCSK9 uptake in PBMCs, thereby causing a decrease in the expression of LDLR. It is expected that lipid clearance via non-drug therapy DFPP has broad applications in the treatment of hyperlipidemia and reduces CVD risk.

ER stress is one of the mechanisms of hyperlipidemia. The ER's primary functions include proper folding of modified proteins, controlling cholesterol production, and storing intracellular Ca^2+^ (33). A previous study indicated that PBMCs ER stress and lipids were strongly associated with coronary artery disease (7, 34). Insults interfering with PBMCs ER function lead to the accumulation of unfolded and misfolded proteins in the ER, which initiates the unfolded protein response (UPR). When the UPR fails to control the level of unfolded and misfolded proteins, ER-initiated apoptotic signaling is induced. When accompanied by high blood lipid levels, excessive ER stress is harmful to the body (34). The ER stress-related protein GRP78 is upregulated in obese animals (33). GRP78, a signature protein of ER stress, can bind to ER stress factors to maintain the ER in a non-stress state under normal conditions (35). Protein kinase R-like ER kinase (PERK) is the initiating protein of the ER stress pathway, and CHOP is also an essential protein that links upstream and downstream proteins. EIF2α is a critical regulatory protein to control protein synthesis. In ER stress, the accumulation of unfolded/misfolded proteins in the ER will phosphorylate EIF2α, leading to a decrease in overall cell protein expression (35). For the first time, the results obtained here indicated that DFPP might be associated with a decrease in ER stress in PBMCs, indicating that lipid plasmapheresis effectively decreases potential risk factors for CVD.

Hyperlipidemia is closely associated with ROS production. A previous study has shown that ROS are produced when endothelial-like cells are exposed to free fatty acids (36). The production of ROS has been proven to be a significant cause of atherosclerosis (37). In addition, inflammatory molecules and apoptosis-related proteins, such as IL-6, TNF-α, and BAX, were abnormally elevated in patients with hyperlipidemia (36, 37). In an animal study, simvastatin administration attenuated brain oxidative stress during experimental sepsis (38). Consistent with lipid-lowering therapy, the lipid plasmapheresis approach reported here was also associated with decreased ROS production, inflammation, and apoptosis. It is unknown whether reducing PBMCs apoptosis is beneficial to patients with hyperlipidemia. Previous studies indicated that increases in macrophage and endothelial cell apoptosis rates were associated with atherosclerosis (39, 40) whether PBMCs apoptosis reduction is beneficial after lipid plasmapheresis remains to be further studied.

### Study Strength and Limitations

DFPP is a safe treatment means, and it can reduce blood lipid levels significantly within a few hours. However, many lipid metabolic protein changes have not been fully studied after DFPP treatment. For the first time, we observed the immediate changes in lipid metabolism-related protein (CD36, LDLR, and PCSK9) before and after DFPP treatment in PBMCs, as well as the changes of ER stress proteins and apoptosis-related proteins. Through the research group's efforts, the changes of proteins were quickly detected by Western blot, and serum samples were quickly detected by FCM, avoiding the error caused by protein and ROS degradation due to long storage time. Changes of serum and PBMCs after removal of lipid provide a basis for further basic experiments in the next step. DFPP can remove specific plasma components by replacing the adsorption membrane so that it may be one of the critical methods for CVD research in the future.

There are several potential limitations in the study. First, the lack of PCR to detect genes related to lipid metabolism (such as Srebp2, Hmgcoar, and Fas) is one of the limitations in this experiment. Although we detected lipid metabolism-related proteins, RT-PCR detection of genes will be an important technical means for the next experiment. Second, the lack of whole-genome sequencing to indicate the signaling pathway involved in lipid metabolism is another defect of this experiment. In the future, the changes of various signaling pathways will be accurately and comprehensively observed through sequencing. This will be a basis for exploring the potential pathways of DFPP treatment.

## Conclusion

This study demonstrated that DFPP reduced the levels of lipids, lipid-metabolizing proteins, and inflammatory factors in patients with hyperlipidemia. It also reduced the levels of ER stress- and apoptosis-related proteins and ROS in PBMCs. Plasma purification shows promise for the treatment of patients with hyperlipidemia. Lipid plasmapheresis is also inexpensive, easy to perform, safe, and effective.

## Data Availability Statement

The original contributions presented in the study are included in the article/supplementary material, further inquiries can be directed to the corresponding authors.

## Ethics Statement

The studies involving human participants were reviewed and approved by the Ethical Committee approved the study of Shanghai Pudong Hospital (Fudan University, Pudong Medical Center, Shanghai, China). The patients/participants provided their written informed consent to participate in this study.

## Author Contributions

XY and HJ conceived of and designed the research. XZ, YG, ZZ, ZX, and HD wrote the manuscript. XL polished the article. All authors conducted the experiments, analyzed the data, commented on previous versions of the manuscript, read, and approved the final manuscript.

## Conflict of Interest

The authors declare that the research was conducted in the absence of any commercial or financial relationships that could be construed as a potential conflict of interest.
